# Supplementary material for: Antibacterial Activity of Chromomycins from a Marine-Derived Streptomyces microflavus
Source: Mar Drugs. 2020 Oct 21;18(10):522. doi: 10.3390/md18100522 (PMC7588889; doi:10.3390/md18100522)
Supplement: Supplementary file 1 [file marinedrugs-18-00522-s001.pdf]

Supporting Information

Antibacterial Activity of Chromomycins from a Marine-Derived *Streptomyces microflavus*

Eunji Cho [1], Oh-Seok Kwon [2], Beomkoo Chung [1], Jayho Lee [1], Jeongyoon Sun [1], Jongheon Shin [2,*] and Ki-Bong Oh [1,*]

[1] *Department of Agricultural Biotechnology, College of Agriculture and Life Sciences, Seoul National University, Seoul 08826, Korea*

[2] *Natural Products Research Institute, College of Pharmacy, Seoul National University, Seoul 08826, Korea*

Contents

Figure S1. The ^{1}H NMR (400 MHz, chloroform-*d*) spectrum of **1**

Figure S2. The ^{13}C NMR (100 MHz, chloroform-*d*) spectrum of **1**

Figure S3. The COSY (400 MHz, $CDCl_3$) spectrum of **1**

Figure S4. The TOCSY (400 MHz, $CDCl_3$) spectrum of **1**

Figure S5. The eHSQC (400 MHz, chloroform-*d*) spectrum of **1**

Figure S6. The HMBC (400 MHz, chloroform-*d*) spectrum of **1**

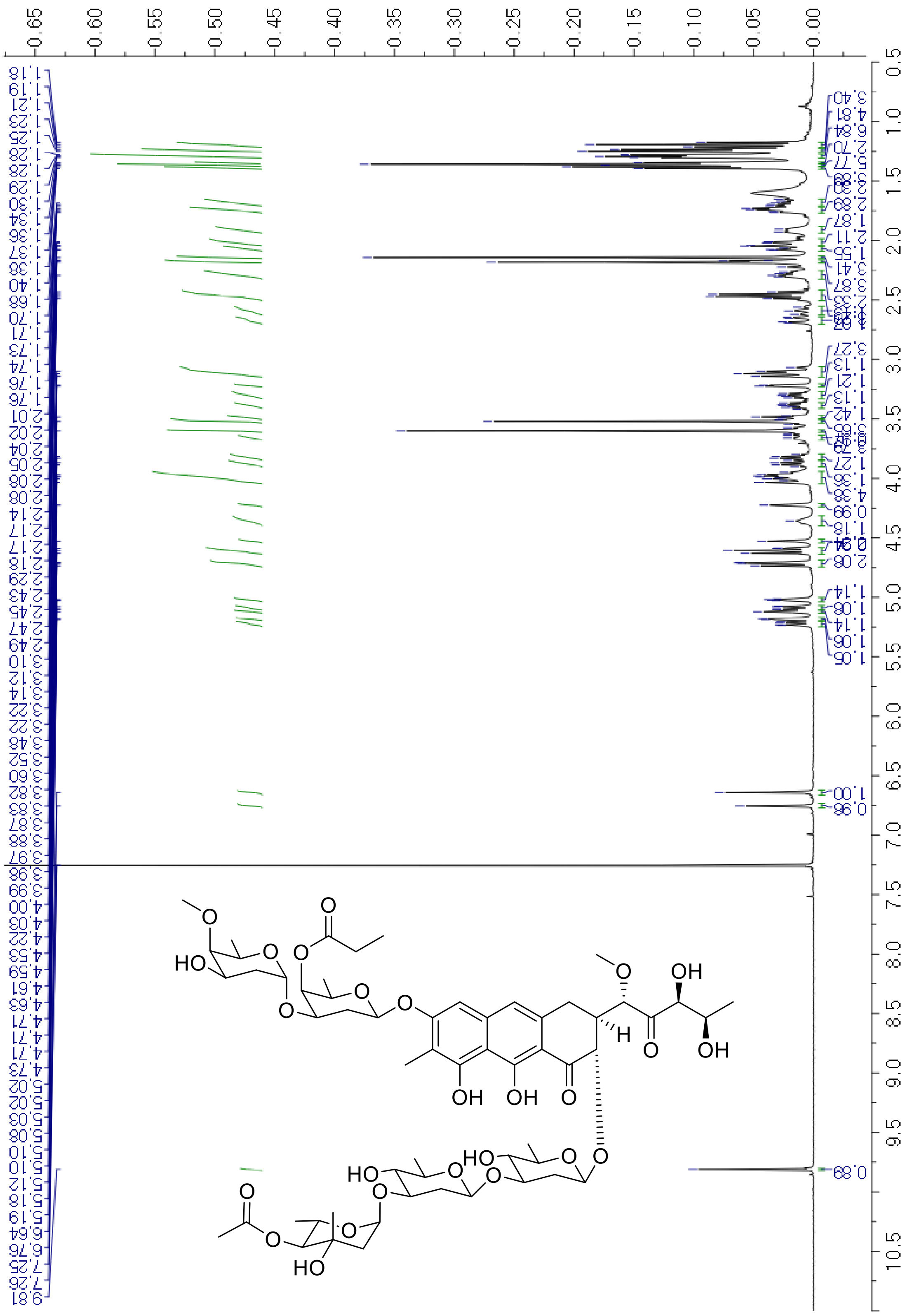

Figure S1. The ^{1}H NMR (400 MHz, chloroform-*d*) spectrum of **1**

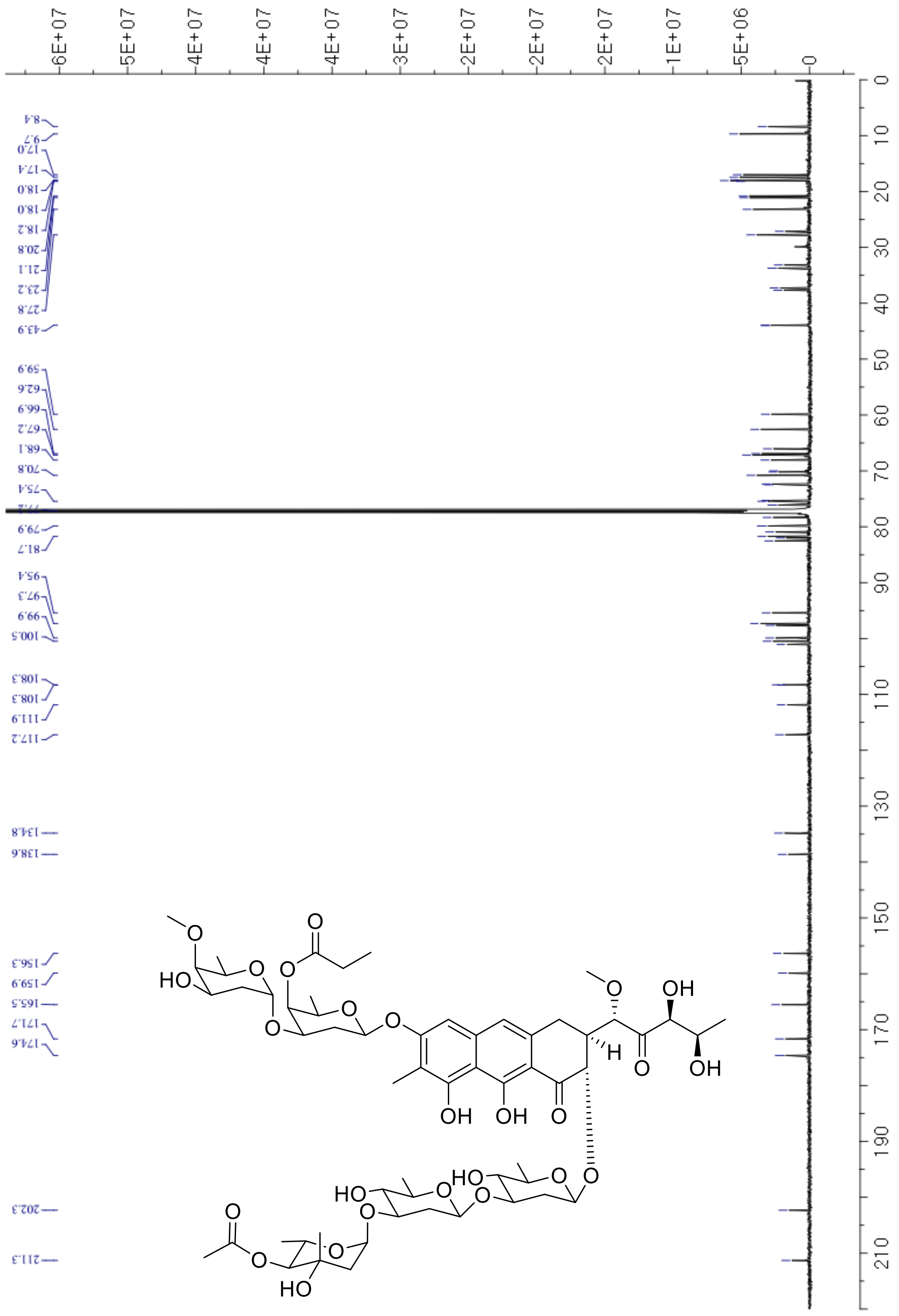

Figure S2. The ^{13}C NMR (100 MHz, chloroform-*d*) spectrum of **1**

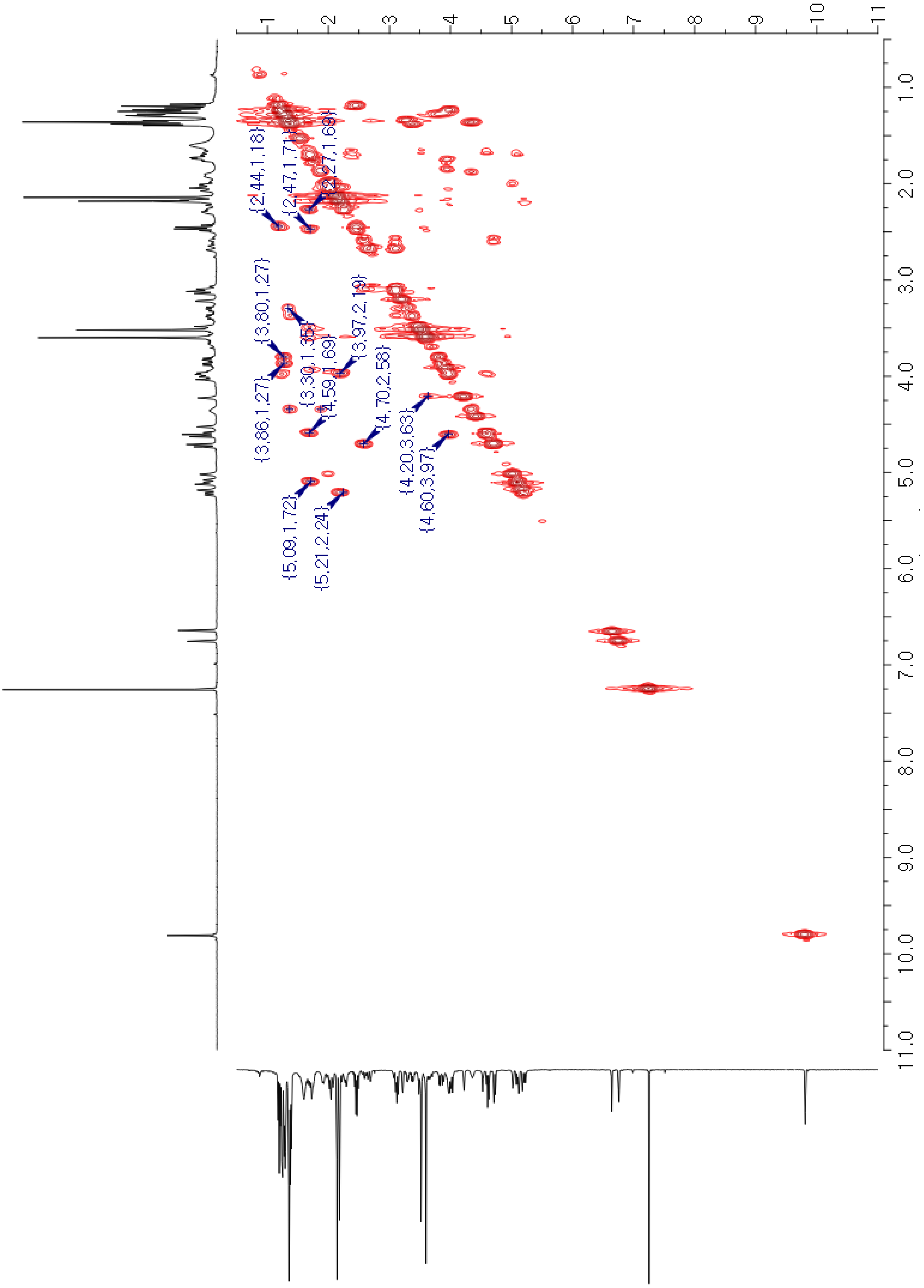

Figure S3. The COSY (400 MHz, $CDCl_3$) spectrum of **1**

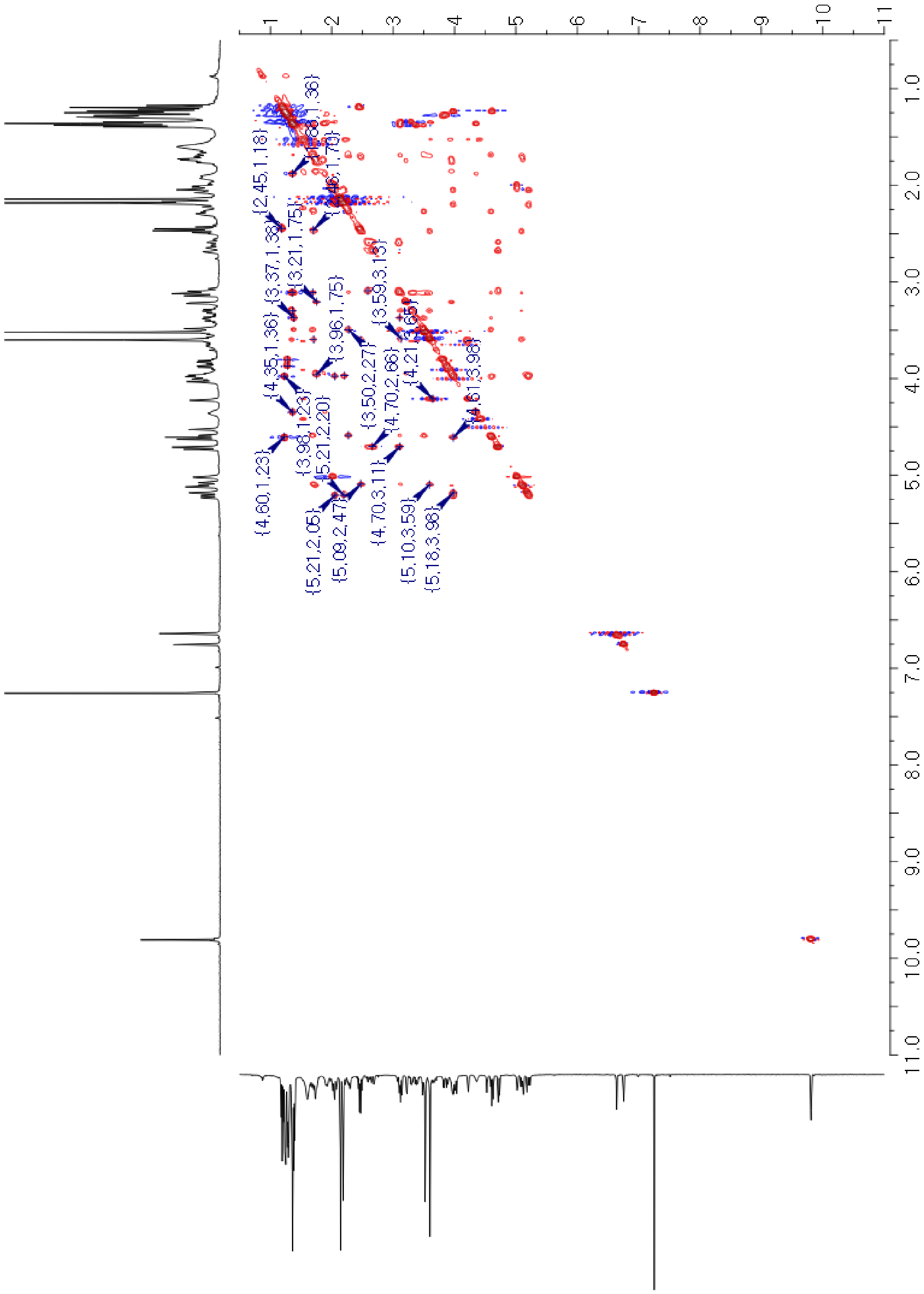

Figure S4. The TOCSY (400 MHz, $CDCl_3$) spectrum of **1**

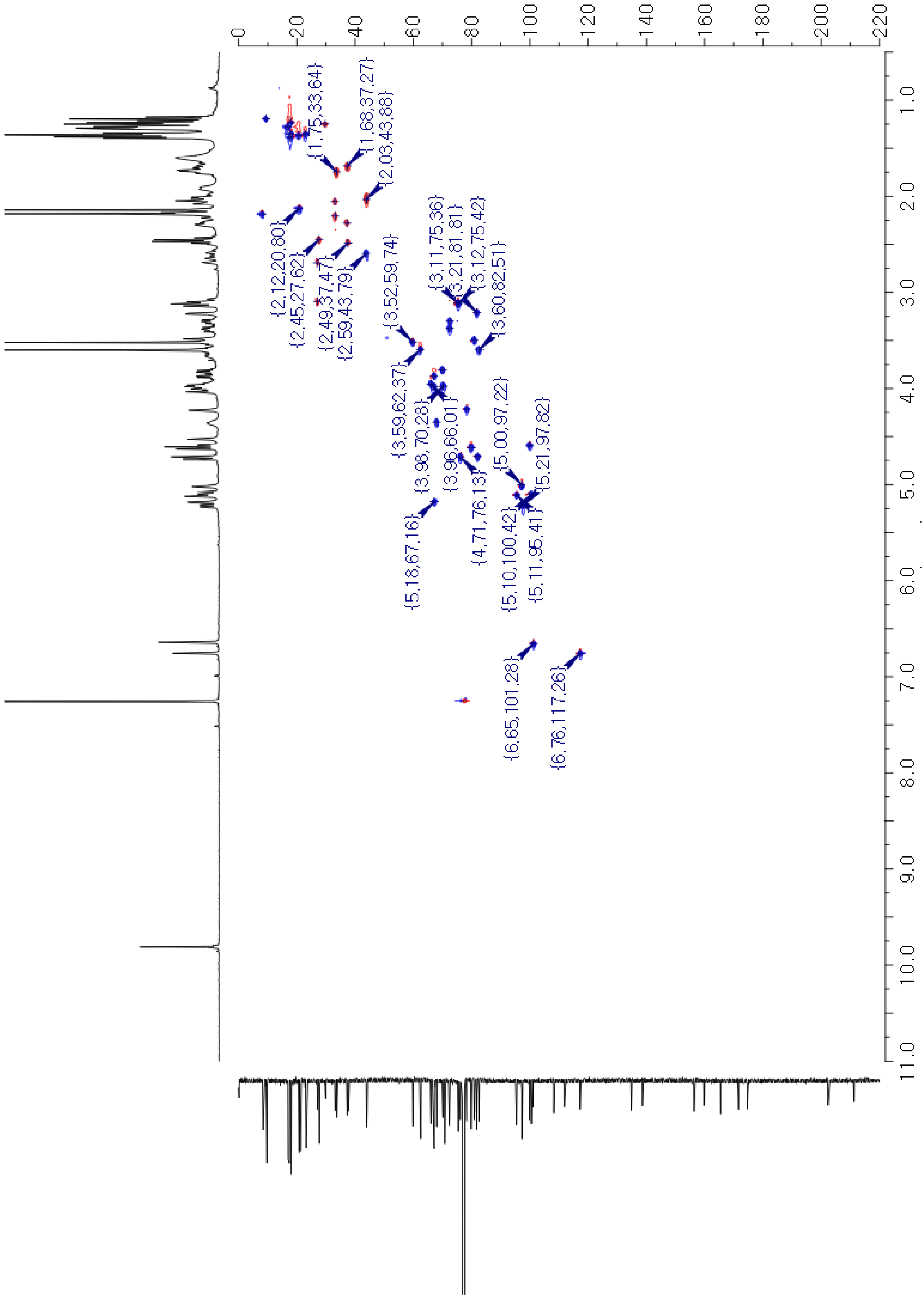

Figure S5. The eHSQC (400 MHz, chloroform-*d*) spectrum of **1**

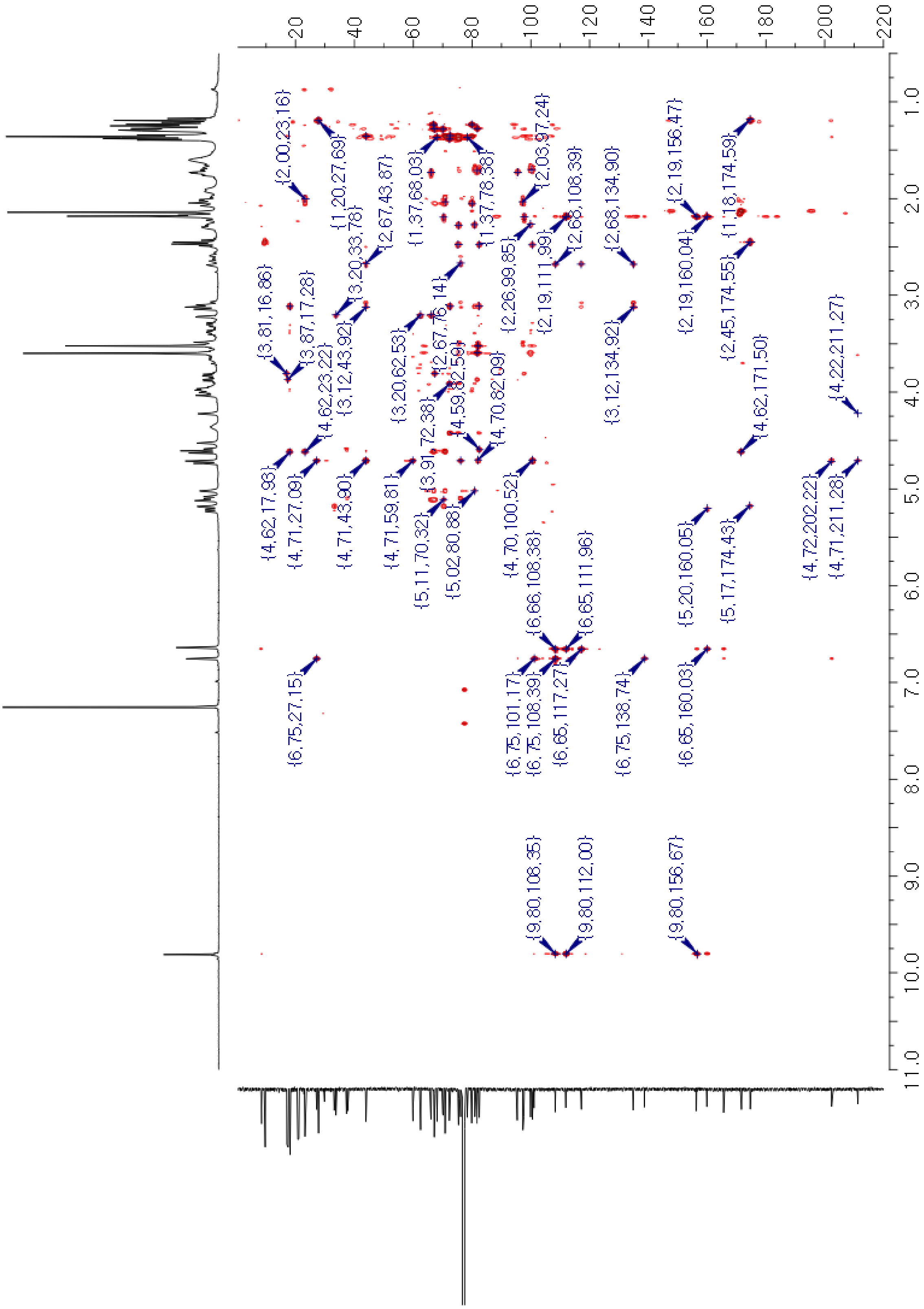

Figure S6. The HMBC (400 MHz, chloroform-*d*) spectrum of **1**
